# Therapy response testing of breast cancer in a 3D high-throughput perfused microfluidic platform

**DOI:** 10.1186/s12885-017-3709-3

**Published:** 2017-11-02

**Authors:** Henriette L. Lanz, Anthony Saleh, Bart Kramer, Junmei Cairns, Chee Ping Ng, Jia Yu, Sebastiaan J. Trietsch, Thomas Hankemeier, Jos Joore, Paul Vulto, Richard Weinshilboum, Liewei Wang

**Affiliations:** 1 Mimetas BV, Leiden, The Netherlands; 2 NIH, Bethesda, Maryland USA; 3 Mayo Clinic, Rochester, Minnesota USA; 4 Leiden University, Leiden, The Netherlands

**Keywords:** Organ-on-a-chip, Personalized medicine, Triple negative, P53 and BRCA1

## Abstract

**Background:**

Breast cancer is the most common invasive cancer among women. Currently, there are only a few models used for therapy selection, and they are often poor predictors of therapeutic response or take months to set up and assay. In this report, we introduce a microfluidic OrganoPlate® platform for extracellular matrix (ECM) embedded tumor culture under perfusion as an initial study designed to investigate the feasibility of adapting this technology for therapy selection.

**Methods:**

The triple negative breast cancer cell lines MDA-MB-453, MDA-MB-231 and HCC1937 were selected based on their different BRCA1 and P53 status, and were seeded in the platform. We evaluate seeding densities, ECM composition (Matrigel®, BME2rgf, collagen I) and biomechanical (perfusion vs static) conditions. We then exposed the cells to a series of anti-cancer drugs (paclitaxel, olaparib, cisplatin) and compared their responses to those in 2D cultures. Finally, we generated cisplatin dose responses in 3D cultures of breast cancer cells derived from 2 PDX models.

**Results:**

The microfluidic platform allows the simultaneous culture of 96 perfused micro tissues, using limited amounts of material, enabling drug screening of patient-derived material. 3D cell culture viability is improved by constant perfusion of the medium. Furthermore, the drug response of these triple negative breast cancer cells was attenuated by culture in 3D and differed from that observed in 2D substrates.

**Conclusions:**

We have investigated the use of a high-throughput organ-on-a-chip platform to select therapies. Our results have raised the possibility to use this technology in personalized medicine to support selection of appropriate drugs and to predict response to therapy in a real time fashion.

**Electronic supplementary material:**

The online version of this article (10.1186/s12885-017-3709-3) contains supplementary material, which is available to authorized users.

## Background

Breast cancer is the most common invasive cancer among women. In the United States, over 200,000 new cases are diagnosed and about 40,000 women die from this disease each year [1, 2]. It is also the most frequently diagnosed cancer among women globally and the leading cause of cancer death, with an estimated 1.7 million cases and 521,900 deaths in 2012 [3]. Based on receptor status, it can be sub-classified into ER+, PR+, HER2+ and triple negative breast cancer. Triple negative breast cancer has the poorest outcome compared to other subtypes [4]. The main FDA approved treatment for primary triple negative breast cancer is still chemotherapy [5]. Although many targeted therapies are being tested in this setting [6], there is a significant need to speed up the pace of drug development and the patient-specific application of these novel drugs in the clinic. Therefore, in this study, we have used triple negative breast cancer cell lines as our models. It is well established that P53 is one of the most commonly mutated genes in triple negative breast cancer and the mutation status of P53 has significant biological implications [7]. BRCA1 mutation is also frequently observed in triple negative breast cancer patients and has significant implications for the therapeutic response to PARP inhibitors and platinum compounds [8–11]. Therefore, the three triple negative cell lines used in the experiments described subsequently were selected based on p53 and BRCA1 mutation status (Table 1), which allowed us to test sensitivity to relevant compounds which are reported to have differential responses when these genetic modifications are present. We envision a possible screening strategy whereby cell cycle inhibiters and other standard chemotherapeutic agents such as, doxorubicin, and taxanes could be tested in vitro prior to therapy selection.

**Table 1:** Triple negative cell lines used in the studies based on their p53 and BRCA1 mutation status

Cell line	BRCA1 status^1^	P53 status^2^
MDA-MB-453	WT	367 30 bp deletion
MDA-MB-231	WT	280 Arg - > Lys
HCC1937	5382insC (fs > 1829X)	306 Arg - > stop

Currently, only a limited number of models are used for therapy selection, predominantly animal based patient-derived xenograft (PDX) cancer models, and in vitro*/*ex vivo models [12]. Animal models, such as mice, are most commonly used to test the efficacies of different therapeutic agents due to their intrinsic complex microenvironments. However, there are profound limitations to their ability to mimic human-specific features. Important factors include general differences between human and animal physiology, metabolism, and tumor cell interactions with the innate immune system, proliferation, metastasis, and the nature of the cells themselves. For years, patient-derived biopsies have been considered a promising tool for predictive therapy selection for breast cancer treatment. Currently, studies are performed in which patient biopsies are engrafted in immune-deficient mice. The PDXs developed in this fashion are grown in mice and subsequently exposed to therapeutic options. However, the long and cumbersome procedure required to develop and test PDXs makes the outcome of these studies only relevant for retrospective studies, rather than as a clinical decision making tools with predictive value. On top of this, there is considerable public and governmental pressure to reduce animal use in experiments.

Direct in vitro culture of patient biopsies and/or tumor resection material may offer a much faster experimental procedure and has been used to predict drug responses using 2D breast cancer cultures [13]. However, the predictive value of these assays has long been questioned. Surface-attached, 2D culture techniques may result in rapid selection of proliferating cells over quiescent cells. Moreover, the artificial environment presented to cells growing on plastic surfaces, initiates uncontrolled (de-)differentiation of cells. In recent years, 3D cell culture, with cells embedded in extracellular matrix (ECM), has rapidly gained popularity as an alternative approach to standard 2D culture and studies. In general, 3D cell culture should offer a more physiologically relevant microenvironment to culture, study and screen cells isolated from biopsies. ECM components allow binding of cell adhesion receptors that influence cell polarity, metabolism, fate and migration [14]. Typical 3D cell culture phenotypes include clustering of cells, lumen formation, reduced proliferation, as well as differentiation. In 2D monolayer cultures, non-malignant and malignant breast epithelial cells often exhibit similar morphologies and doubling times. In contrast, 3D culture assays have been shown to produce phenotypic discrimination between non-malignant and malignant breast epithelial cells [15–17], where non-malignant epithelial cells form polarized, growth-arrested tubular-like structures when grown in 3D ECM gels [18]. In contrast, malignant cancer cells form disorganized and proliferative spheroids [19].. In addition, it is known that ECM-embedded culture of cells from breast cancer biopsies yields 3D spheroids that form milk secretion channels [20]. Sung and coworkers investigated human mammary fibroblasts (HMFs) cultured in 2D and 3D and their effects on the invasive phenotype transition of breast cancer cells. HMFs cultured in 3D induced a more invasive phenotype of the breast cancer cells than observed in their 2D–cultured counterparts. HMFs in 3D also produced more signaling molecules such as fibroblast-derived HGF that are essential for the progression of breast cancer cells from a non-invasive to an invasive phenotype [21]. These studies strongly suggest that engineered tissue models that incorporate 3D culture in tumor relevant ECM and co-culture with tumor stroma, represent promising and relevant tools that allow modeling of the tumor microenvironment in vitro [12, 22, 23]. Notwithstanding a great deal of evidence for the superiority of 3D culture techniques over 2D, implementation of 3D culture on a large scale is still cumbersome. The availability of tissue material is often limiting, particularly when a range of different conditions need to be tested. Thus, biopsy samples may need to be expanded as 3D spheroids to ensure that enough cells are available for a robust compound screen. Also, experimental procedures can be cumbersome, particularly relating to the readout and data interpretation of often highly non-uniform cultures.

Microfluidics-based cell culture techniques have generated tremendous interest in recent years. The marriage between cell culture and microfabrication techniques holds the promise of a precise spatial and temporal control of the microenvironment and incorporation of mechanical stimuli such as fluid flow as experienced by cells in vivo. This approach may also overcome issues associated with traditional 3D culture methods such as non-uniformity and the limited availability of tissue materials. Such platforms, also typically known as “organs-on-a-chip”, enable the integration of co-culture, perfusion flow, gradients (e.g. chemotaxis), and mechanical strains and could ultimately lead to mimicry of crucial aspects of human organ functionality in an in vitro setting [21, 24, 25]. For therapy selection, crucial benefits of microfluidics-based techniques include low volumes, the ability to engineer the microenvironment through ECM-embedded culture, perfusion flow and co-culture of selected tissues and cell types. The low culture volumes are of utmost importance, since the amount of material available from patient biopsies may be minimal, whereas thousands of compounds and combinations need to be screened to tailor an optimal and effective therapy. Recently, the value of more physiologically relevant microfluidic platforms for studying breast tumor processes such as migration, invasion, extravasation, co-culture (with stromal or/and endothelial cells) or/and mechanical stimuli such as interstitial flow [23, 26–31] has been demonstrated. These 3D cell culture organ-on-a-chip systems permit high-resolution, real-time molecular imaging to provide insight into a drug’s mechanism of action, as well as mechanism of toxicity. Similar to other 3D non-microfluidic culture models, studies using these platforms have also demonstrated differential functionalities and responses to drug exposure in comparison to traditional 2D plastic plate cultures and they are better at mimicking in vivo conditions. However, their throughput is typically limited to one or a few cultures, which renders them unfit for application to therapy selection. Recent advances have also been made in high throughput 3D culture microfluidic platforms more in line with traditional 96- and 384-well 2D high throughput toxicity screens, as approaches in molecular and cell biology and compound discovery often require testing of multiple conditions with controls, replicates and dilutions in a single experimental setup [32]. A microfluidic system for 3D cell culture was developed and used by Montanez-Sauri et al. [27] as a proof-of-concept to screen for the effect of ECM composition and MMP inhibition on the phenotype, behavior, and proliferation of T47D breast carcinoma clusters in monoculture and co-culture configurations. It was also employed to screen and detect inter-individual heterogeneity of paracrine interactions between T47D breast carcinoma cells and breast carcinoma-associated fibroblasts of various grades or normal mammary fibroblasts isolated from breast carcinoma tissue samples and adjacent normal mammary gland tissue from patients [33]. Trietsch and coworkers developed a stratified platform that is incorporated in a microtiter plate format that is fully compatible and easy to handle with standard automation and high-content screening equipment [24]. This platform, known as the OrganoPlate®, has been used for iPS neuron differentiation [34] [35] and liver spheroid culture under perfusion [24, 36].

In this paper, we employ a microfluidic organ-on-a-chip platform based on the standard 384-well plate format and we study its potential applicability for breast cancer therapy selection. Specifically, we optimizes seeding densities, ECM composition and biomechanical conditions for a series of 3 distinct breast-cancer cell lines. Subsequently, we exposed the cells to a series of anti-cancer agents and compared the responses to those observed in 2D cultures. As a proof of concept for the use of patient material we used PDX-derived human cancer cells to determine their cisplatin sensitivity in 3D in vitro culture. Finally, we present our view on the potential usage of microfluidics-based 3D cell culture models for guiding personalized therapy selection in the clinic.

## Methods

### Cell culture

The triple negative breast cancer cell lines MDA-MB-453, MDA-MB-231 and HCC1937 were obtained from the American Type Culture Collection (HTB-131, HTB26 and CRL-2336 respectively, ATCC, Manassas, VA). Cell lines were selected based on their different BRCA1 and P53 status (Table 1). MDA-MB-453 and MDA-MB-231 were maintained at 37 °C, 100% air, and HCC1937 at 37 °C, 5% CO_2_. MDA-MB-453 and MDA-MB-231 were cultured in L15 medium (ATCC) supplemented with 10% foetal calf serum (FCS, ATCC) and 5% penicillin/streptomycin (p/s, 100 units penicillin/mL, 100 μg streptomycin/mL), HCC1937 in RPMI-1640 (ATCC), 10% FCS and 5% p/s, all according to supplier’s protocol (Additional file 1 Table S1).

For 2D culture, cells were seeded on tissue culture grade plastic T75, 96-well flat bottom plates (Corning, Amsterdam, The Netherlands). For 3D cell culture, cells were trypsinized, pelleted and resuspended at the indicated concentration in the appropriate extracellular matrix (ECM). Matrigel® (Corning) was used at 9 mg/mL, and BME2rgf (Amsbio, Abingdon, UK) at 15 mg/mL. Aliquots of both were thawed on ice 1 day prior to seeding. Collagen type I rat tail (Amsbio) was neutralized with Na_2_CO_3_ (Sigma-Aldrich Chemie B.V., Zwijndrecht, the Netherlands) and buffered with 100 mM HEPES (Sigma) to a final concentration of 4 mg/mL, prior to resuspending the cells.

PDX-tumors were generated according to previously described protocol [37] and single tumor cell suspension generation from PDX-tumors were derived as follows. Tumor cells from two triple negative breast cancer PDX were isolated using the human Tumor Dissociation Kit (Order no. 130–095-929, Miltenyi Biotec). Briefly tumors were cut into small pieces of 2–4 mm, then transfered into the gentleMACS C Tube and run the 7C_h_TDK3 program according to manufacturer’s protocol. Next the tubes were centrifuged to collect the sample material, and washed with washing buffer. Mouse Cell Depletion Kit (Order no. 130–104-694, Miltenyi Biotec) was used to enrich human cells. Specifically, cell pellet was resuspended in buffer, 20 μL of the Mouse Cell Depletion Cocktail added and incubated for 15 min at 4 °C. Then magnetic separation with LS Columns was performed to collect human cells. PDX-derived cells were cultured in DMEM (Sigma) supplemented with 10% FCS, 1% glutamax (35.050.061 Gibco), 1% sodium pyruvate (11.360.070 Gibco), non-essential amino acids (11.140.076, Gibco) and 1% p/s (15.140.122, Gibco) at 37 °C, 5% CO_2_.

### 3D plate loading

**Fig. 1:**
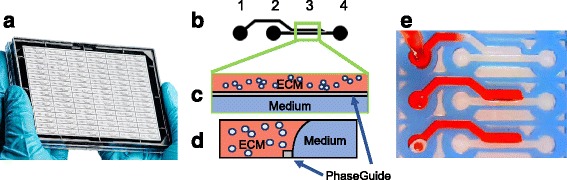
Microtiter cancer-on-a-chip plate for 3D breast cancer therapy response testing. **a** Photo of OrganoPlate® platform consisting of 96 perfusable microfluidic chambers in parallel. **b** Closeup, (**c**) top and (**d**) side view of an individual chamber consisting of an ECM channel and a Medium channel. Cells are premixed into a gel solution, loaded into the ECM channel by capillary action and allowed to polymerize before the introduction of medium into the adjacent Medium channel for culture. PhaseGuide™ allows the gel solution to be pinned during the loading and polymerization step, thereby allowing support-free and unhindered exchange with the medium. **e** Photo demonstrates the filling of the ECM channel using a red dye

200 μm and 400 μm 2-lane OrganoPlates® consisting of 96 microfluidic chips in parallel were obtained from Mimetas B.V. (Leiden, the Netherlands, Fig. 1a-b and Additional file 1 Figure S1). In each chip, 1 or 2 μL of cells (200 μm and 400 μm plate respectively) resuspended in liquid ECM were patterned by the PhaseGuide™ alongside an empty channel in the microfluidic chip by capillary force (Fig. 1b-e). Plates were placed under regular culture conditions to allow gelation of the ECM (Matrigel® and BME2rgf for 15 min, collagen I for 30 min). After gelation, the remaining empty channel was filled with media which could be passively perfused by the levelling of two connected wells (total 100 μL medium). By placing the plate on a modified rocker platform at 7 degrees with an 8 min interval, a continuous, bi-directional average flow of 1 μL/min was achieved. For static conditions, plates were placed flat in the incubator with equal volumes in medium wells. PDX-derived cells were seeded at 1*10^7^ cells/mL in a 400 μm 2-lane plate. Medium was refreshed three times a week in the OrganoPlate®, as for regular 2D culture.

### Image analysis for cell viability and immunohistochemistry

Cell morphology was imaged with phase contrast microscopy twice a week on a high content imaging system the ImageXpress Micro (Molecular Devices, Sunnyvale, CA). Viability of 3D cell cultures was assessed with fluorescent live/dead stain (calcein-AM, NucBlue® (Hoechst), NucRed® Dead 647, Life technologies, Bleiswijk, the Netherlands). Cultures were incubated for 30 min with medium containing 4 μM calcein-AM and NucBlue® and NucRed® at 2 drops/mL. Stained cells were imaged with the ImageXpress Micro XLS (Molecular Devices) and analysed with Image J software (NIH, Bethesda, MD) [38].

Cells were fixed for 10 min with 3.7% formaldehyde (Sigma) in PBS (phasephate-buffere saline, Life technologies #20012068). Cells washed twice for 5 min with PBS and permeabilized with 0.3% Triton X-100 (Sigma # T8787) in PBS for 10 min. After washing with 4% FCS in PBS, cells were incubated with blocking solution (2% FCS, 2% bovine serum albumin (BSA) (Sigma # A2153), 0,1% Tween 20 (Sigma # P9416) in PBS) for 45 min. Subsequently, cells were incubated with primary antibody (rabbit –a-phospho-H2A.X, Cell Signalling 9718S) for 60 min, washed 3 times with PBS, incubated with secondary antibody (goat-a-rabbit-alexa488, Life technologies, A32731) for 30 min and washed 3 times with 4% FCS in PBS. After nuclear stain (NucBlue®) cells were stored in PBS at room temperature and imaged with the ImageXpress Micro XLS.

### RealTime-Glo™ and CellTiter-Glo® viability assay

Optimal seeding densities for toxicity exposures for both 2D (96 well plate) and 3D (OrganoPlate®) cultures were determined using the luminescent, non-lytic, RealTime-Glo™ assay (Promega, Leiden, the Netherlands) according to the manufacturer’s protocol. Upon replacement of medium in medium wells with 1X RealTime-Glo™ reagent, measurement of the luminescent signal was started in time on a Fluoroskan Ascent FL microplate reader (Life technologies). For 3D cultures the luminescent signal of the four wells aligning with the microfluidic chip were combined for calculations.

PDX-derived cell viability in 3D cultures upon cisplatin exposure was determined using the luminescent CellTiter-Glo® assay (Promega). Medium was replaced by 1× solution and incubated for 45 min on the rocker at 37 °C, 5% CO_2_ after which luminescent signal was measured.

### Toxicity studies

For toxicity assays, olaparib (Sanbio, Uden, The Netherlands) and paclitaxel (Sigma) were dissolved in DMSO (Sigma). The final DMSO concentration in the medium during exposure was 0,4%. Cisplatin (Sigma) was dissolved prior to use in medium. Cells were seeded in 2D and 3D 1 day before the start of exposure (*t* = 0). Prior to exposure, baseline viability was determined by RealTime-Glo™. Values were used to correct for variation in seeding density at *t* = 0. MDA-MB-231 and MDA-MB-453 (BRCA WT, P53 mutant) cell lines were exposed to increasing concentrations of cisplatin for 48 h. HCC1937 (BRCA mutant) was exposed to olaparib and paclitaxel for 72 h. After exposure, viability was measured once again with RealTime-Glo™.

### Statistical analysis

**Fig. 2:**
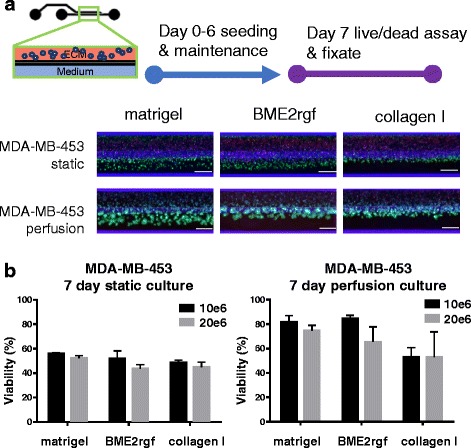
Culture optimization in the microtiter microfluidic platform. Up to 96 multiple conditions such as seeding density, ECM composition, cell types and perfusion can be investigated concurrently. Breast cancer cell line MDA-MB-453 was seeded in three different ECM compositions at two different densities and maintained for 6 days before assessment with a live/dead assay (Calcein AM - green/NucBlue® (Hoechst) - blue/NucRed® (propidium iodide) - Red). Scale bar = 400 μm. **a** Epifluorescence microscopy images showing different morphologies and viabilities of MDA-MB-453 in Matrigel®, BME2rgf and collagen I under static and perfusion conditions at a seeding density of 10*10^6^ cells/mL. **b** Graphs quantifying the effect of ECM (Matrigel® vs BME2rgf vs collagen I), seeding density (10*10^6^ cells/mL, black, vs 20*10^6^ cells/mL, grey), and static vs perfusion culture on the viability (represented as % of total cells) of MDA-MB-453 cells. Total cell number was determined by nuclear count (Hoechst staining). Total number of dead cells was determined by positive propidium iodide staining. Viable cells was set at total cell number minus dead cell count

**Fig. 3:**
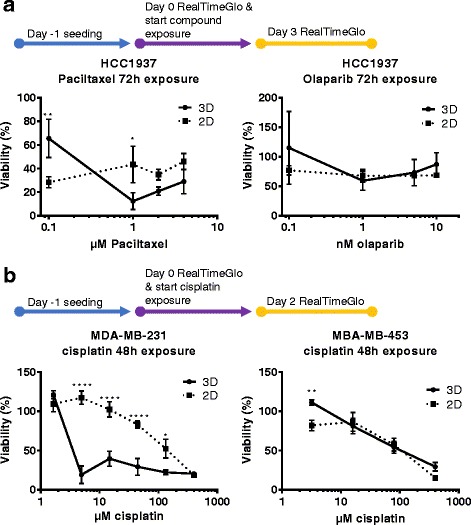
Screening studies of breast cancer cell lines in microfluidic culture**. a** For paclitaxel and olaparib studies, HCC-1937 were seeded 3D in the OrganoPlate® and 2D on tissue culture grade plastics 96-well flat bottom plates, cultured for 1 day before 72 h exposure with compounds at the specified concentrations. Viability (as % of total cells) was quantified using an optimized RealTime-Glo™ cell viability assay. (B) MDA-MB-231 and MDA-MB-453 were seeded for 24 h similarly and exposed to cisplatin at various concentrations for 48 h. Symbols: * *P* ≤ 0.05, ** *P* ≤ 0.01, *** *P* ≤ 0.001, **** *P* ≤ 0.0001 using Tukey’s multiple comparison test (See supplementary documents for *p*-values and more detailed analysis)

All experiments are performed at least in triplicate or as indicated. 2-way ANOVA with Tukey’s multi-comparison post-test was performed on data using Prism 6 (GraphPad Software, Inc., La Jolla, CA). Due to the numerous comparisons, *P*-values and significance difference between tested conditions were presented separately in supplementary Additional file 1 Table S2 and Additional file 1 Table S3 from their graphs in Figs. 2 and 3.

## Results

### Microfluidic platform for 3D breast tissue culture

Figure 1a shows the OrganoPlate® platform. This chip that is manufactured and marketed by Mimetas, is a 384 microtiter well plate that is modified on the bottom with microfluidic channel structures. These channel structures are construed using a polymer microfluidic layer that is sandwiched between two 175 μm glass plates. In this paper, a so-called two-lane device is used for which 96 networks are present on one plate. Each microfluidic network (Fig. 1b) interacts with 4 wells of the 384 well plate: one well for ECM addition, one well for inserting growth medium in the perfusion channel, one well as an outlet for the perfusion channel and one well for optical interrogation of the microfluidic channel. The central channel underneath the readout well is subdivided into two parts by a PhaseGuide™. The PhaseGuide™ is a thin ridge on the bottom of the microfluidic channel that acts as a pinning barrier for incoming fluids [39]. Meniscus pinning is based on the principle that a sudden change in geometry requires additional energy for a liquid-air meniscus to advance beyond a barrier. The PhaseGuide™ height in this design is 30 μm: one fourth of the microfluidic channel height. Figure 1e shows the filling of the first lane of the network with an ECM gel. The bottom two networks have already been filled with ECM gel that remains pinned on the PhaseGuide™. Once the gel is gelated, the second lane is filled with growth medium (see Fig. 1c-d). Since gel stratification is achieved by meniscus pinning, there is no artificial membrane between the perfusion lane and the ECM gel. Flow of growth medium is achieved by leveling between reservoirs 2 and 4. By placing the platform on an interval rocker, the platform can be placed at an angle to assure leveling in the first direction. By changing the angle of the platform, the direction of fluid flow is reversed.

### 3D triple negative breast cancer model optimization

Figure 2a shows MDA-MB-453 cells seeded in three ECM matrices (Matrigel® vs BME2rgf vs collagen I), under static and perfused conditions, and at two different seeding densities (10*10^6^ cells/mL, black, vs 20*10^6^ cells/mL, grey). In all 6 fluorescent images, the ECM gel lane is the top-lane. Cells were cultured for 7 days and stained with a live cell marker (calcein-AM), a dead cell marker (NucRed®, propidium iodide) and Hoechst (NucBlue® DNA stain, Hoechst) for total cell number.

Although cells are suspended only in the ECM gel, they appear to be present also in the perfusion lane. This is due to meniscus stretching: The ECM gel upon pinning on the PhaseGuide™ stretches along the top-side of the chamber, thus extending into the perfusion flow lane (see also Fig. 1d). Interestingly, cells seem to cluster more in the overhanging part of the meniscus than in the ECM channel, as indicated by the higher fluorescence intensity. Possibly the presence of oxygen and nutrient-rich medium flow and a thinner layer of ECM gel induces this phenotype. This hypothesis is further supported by the static experiments, in which cells in the ECM compartment are no longer viable, while slight survival is still observed for the overhanging part of the gel meniscus. Experimental results are depicted quantitatively in the graphs shown in Fig. 2b. A striking difference in survival is observed between cultures under rocker perfusion and under static conditions. Under the first condition, survival rates are up to 80% for cells in Matrigel® and BME-rgf for lower seeding densities, while survival is significantly lower in collagen I. Under static conditions cell survival falls under 60% in all cases. The experiment clearly underlines the necessity of perfusion flow for optimal survival. The flow not only provides continuous refreshment of growth medium, but also removes waste metabolites, supplies oxygen and induces interstitial flow. A higher seeding density showed a small decrease in viability in most conditions, likely due to reduced nutrient availability.

Similar flow-based improvement of viability results were obtained for the MDA-MB-231and HCC1937 cell lines (data not shown).

Strikingly, the three cell lines showed quite different morphologies. Whereas MDA-MB-453 shows clustering of cells, HCC1937 seems to display a more invasive behavior, occupying much more of the perfusion flow channel. MDA-MB-231 preferentially forms a barrier tissue in collagen I gel (Additional file 1 Figure S1). Optimal conditions for all three cell lines in terms of survival rate, were obtained in Matrigel® at a seeding density of 10*10^6^ cells/mL, under perfusion flow conditions.

### 2D vs 3D compound exposure of breast cancer model

The optimized conditions obtained above were used for testing dose response to chemo-therapeutic agents. An enzymatic activity assay (Real-TimeGlo™) was used as a measure of viability. The optimization of this assay is described in the supplementary data and includes further seeding density optimization showing a linear relationship between cell number and enzymatic activity between 1*10^3^ and 1*10^4^ cells seeded per chip (Additional file 1 Figure S2).

First, HCC1937 were exposed to paclitaxel and olaparib (Fig. 3a). Both 2D and 3D cultures showed a loss of viability to paclitaxel, with a more striking effect in 3D. The maximum effect in 2D is already achieved with the lowest concentration tested, where for the 3D culture this is reached at 1 μM. On the contrary, olaparib showed hardly any effect in either 2D or 3D cultures, as was observed previously [40]. The combination of olaparib and paclitaxel results in a small, but significant increase in viability as compared to paclitaxel in the 3D cultures.

A much more striking difference in response was found when exposing MDA-MB-231 to cisplatin (Fig. 3b). 3D cultures responded to the drug addition at much lower concentrations, while 2D cultures were only affected at higher concentrations in a more dose dependent manner. This difference was much less pronounced for MDA-MB-453, which showed a very similar response in 2D and 3D culture.

**Fig. 4:**
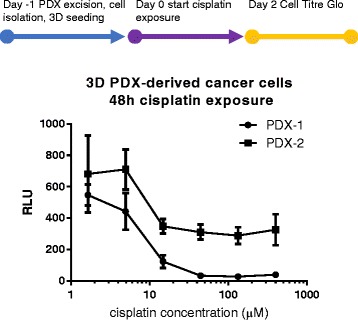
Cisplatin exposure of PDX-derived human breast cancer cells in 3D microfluidic culture. Human cancer cells from two different breast cancer PDX avatars were isolated and seeded in 3D in the OrganoPlate® 1 day prior to 48 h cisplatin exposure. Culture viability was quantified using the luminescent CellTiter-Glo® cell viability assay. IC50 were determined based on nonlinear fit of the dose response range as 8,1 μM and 14,8 μM for PDX-1 and PDX-2 respectively

Finally we tested the compatibility of the 3D microfluidic culture platform with the 3D culture of fresh patient material. Primary tumor cells were isolated from PDX avatars of two patients and seeded in Matrigel® in the OrganoPlate®. The dose response curve for 48 h exposure to cisplatin was obtained, yielding a 8,1 μM and 14.78 μM IC50 for PDX-1 and PDX-2 respectively (Fig. 4).

## Discussion

**Fig. 5:**
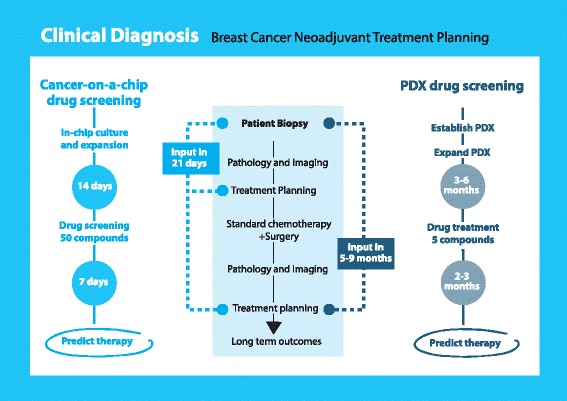
Outlook: Work flow for Patient derived xenograft (PDX) vs cancer-on-a-chip drug screening. Compared to PDX drug screening, the compact OrganoPlate® platform is expected to reduce assay time and space, and increase the throughput of screened compounds, leading to improvements in cancer treatment planning and personalized medicine for individual patients

There will be wide application of the type of technology studied here in the future. A major goal for developing this technology is to help direct and speed up the selection of therapies by predicting responses based on drug testing in 3D cultures grown directly from human tumor samples. In current therapy selection methods, most in vitro drug screens are performed in 2D culture but the results have often proved less than optimal. Alternatively, patient derived xenograft models have become a popular in vivo model to capture human tumor heterogeneity. However, PDX models are expensive and labor-intensive to develop. It also takes many months for tumors to develop in mice. These disadvantages limit the use of PDX models in a real-time setting to help predict treatment response and test drugs for patients. Here, we propose the use of 3D microfluidic models as a new approach to overcome the issues faced with existing 2D and PDX models (Fig. 5). A major goal for developing this technology is to create 3D physiologically-relevant cultures of human tissue which might allow improved and real-time selection of therapy and prediction of response based on drug testing using 3D culture grown directly from human tumor samples. This approach would significantly shorten the timeline for drug screening. Equally important, results from the 3D culture screenings are likely to be closer to in vivo results, rendering them more accurate for directing the selection of appropriate drugs and predicting response for individual patients, thus, achieving personalized therapy.

In this report, we introduce a microfluidic platform for ECM embedded tumor culture under perfusion as an initial study to investigate the feasibility of adapting this technology to therapy selection. Interestingly, drug responses in these cultures were different from 2D cultures, and also different among breast cancer subgroups. The platform has the advantage that it comprises 96 culture chambers that require only very small amounts of primary tumor material, approximately 10.000 cells per data point. This makes the platform promising for studying drug response directly with patient biopsy material, rather than first requiring expansion of the tumor in xenografts or on plastic. From the results shown in this paper, we observed that the cell lines showed improved viability when cultured under perfusion flow conditions. Furthermore, the different triple negative subtypes showed different morphologies among cell types, and within a cell type depending on the ECM composition. MDA-MB-453 showed a grape-like morphology, as previously reported [41], with cluster size depending on the ECM and the distance from the medium perfusion channel. MDA-MB-231 displayed a stellate phenotype in Matrigel®-type ECMs [41], with a switch to a more boundary-like morphology when seeded in collagen I, indicating a possible modulation of the epithelial-to-mesenchymal switch depending on the ECM composition used.

Differences in drug response between cancer cells cultured in 2D and 3D have been reported previously with, in general, a higher resistance in cells cultured in an ECM [17]. Here we observed increased sensitivity of the MDA-MB-231 cell line embedded in Matrigel® when exposed to cisplatin at concentrations between 0,5 and 400 micromolar. Stable in vivo plasma concentrations of cisplatin are generally in the low micromolar range [42], thus our 3D model of MDA-MB-231 accurately predicts sensitivity at a physiologically relevant dose. Cisplatin IC50 values determined for PDX-tumor isolated cells cultured in vitro in 3D are within the same relevant dose range.

The difference between 2D and 3D response is quite striking as cells in 3D generally show slower proliferation rates compared to 2D culture, and are expected to be less sensitive to anti-mitotic agents. However, Huyck et al. also showed higher sensitivity of MDA-MB-231 cells embedded in collagen to the thymidine synthesis inhibitor 5-fluoruracil [43]. Drug response in 3D might be further tuned by varying the composition of the ECM [17]. While initial growth factor concentrations in various ECM gels may impact survival of seeded cells, after a day or so of culture, growth factor contribution to cancer cell growth rate and viability following drug treatment is likely determined by the far higher concentration of growth factors originating from serum in cell culture media.

Apart from a decrease in viability we could detect an increase in the DNA damage marker phospho-H2A.X when exposing the MDA-MB-231 to cisplatin in 3D (Additional file 1 Figure S3) [44].

The difference in drug response between various cancer subgroups also further validates the need to build personalized models for patients, revealing the importance of using cell lines to create additional 3D models that might be more predictive to study breast cancer-related processes such as metastasis, invasion, and to screen compounds for therapeutic intervention.

A significant amount of work lies ahead in which we will test and evaluate primary patient biopsies in the OrganoPlate®, assess their longevity and retrospectively compare drug response to clinical outcome. Critical aspects to take into account will include stroma-tumor interaction. Furthermore, the model could be enhanced by including vascularity and aspects of the immune system. The OrganoPlate® platform is particularly suited for such complex co-cultures, as various cell types can be arranged in orderly lanes, one next to the other, without the usage of artificial membranes. The challenge, however, may lie in the compatibility of the medium and matrix with all cell-types needed for the co-culture. Beyond optimization of cell culture conditions, the models need to be sufficiently robust and validated in order to improve their usability as an effective screening tool. While the current OrganoPlate® platform may not totally capture the in vivo complexity of various mechanical stimuli, cell-types, and interactions with other organs, it is likely to offer a better predictive model than conventional 2D models while being easier and less time consuming to set up for the biologists and clinicians in comparison to models incorporating advanced features or animal models.

Finally, we have described our vision for how these organ-on-a-chip models may be implemented in the clinic, raising the possibility of real-time screening of compounds based on patient genetic profiles to achieve personalized medicine.

Thus, based on our proposed workflow, application of 3D cultures isolated and grown directly from human biopsies or surgical samples would significantly shorten the timeline of drug screening and results from the 3D culture screening might be closer to in vivo results that would be more accurate to select appropriate drugs and to predict response for individual patients (Fig. 5). Other application of this technology could be the use of 3D culture in drug development to help screen compounds. We know that 3D culture has significant advantages compared with 2D culture and that it may offer a more accurate predictive tool for in vivo response.

## Conclusions

In this study, we developed the basis for a 3D breast cancer screening platform using a microfluidic device. The OrganoPlate® allows the simultaneous culture of 96 perfused micro tissues, using limited amounts of material, enabling drug screening of patient-derived material. We showed that 3D cell culture viability is improved by the constant perfusion of the medium. Furthermore, it was demonstrated that the drug response of triple negative breast cancer cells can be attenuated by culture in 3D. Finally we showed compatibility of the platform with fresh dissected tumor material in a dose range exposure.

Even though this technology is still in its infancy, our results have already raised the possibility of using this technology in personalized medicine to help select appropriate drugs and to predict response to therapies in a real time fashion. Once fully developed and validated, we believe that it will also help drug development in a more cost effective fashion that has the potential to achieve greater accuracy.

## Additional file

Additional file 1: Table S1. Culture conditions for the breast cancer cell lines used. **Table S2.** Statistical analysis of culture condition studies in Fig. 2 using Tukey’s multiple comparisons test from GraphPad version 6. **Table S3.** Statistical analysis of compound screening studies in Fig. 3 using Tukey’s multiple comparisons test generated from GraphPad version 6. **Figure S1.** Raw data: Array of phase contrast, fluorescent live/dead images of the breast cancer subgroups MDA-MB-453, MDA-MB-231 and HCC 1937 cultured in 3D perfusion culture. **Figure S2.** Realtime-Glo™ (RTG) assay optimization for HCC1937 and MDA-MB-231 in the OrganoPlate® for day 0 and day 7 culture. **Figure S3.** MDA-MB-231 were cultured for 1 day in matrigel prior to 48 h 100 μM cisplatin exposure in the OrganoPlate®. (PPTX 82839 kb)

## Abbreviations

ECM: Extracellular matrix
FCS: Fetal calf serum
HMF: Human mammary fibroblast
PDX: Patient-derived xenograft
