# Supplementary material for: Therapy response testing of breast cancer in a 3D high-throughput perfused microfluidic platform
Source: BMC Cancer. 2017 Nov 2;17:709. doi: 10.1186/s12885-017-3709-3 (PMC5668957; doi:10.1186/s12885-017-3709-3)
Supplement: Additional file 1: Table S1. — Culture conditions for the breast cancer cell lines used. Table S2. Statistical analysis of culture condition studies in Fig. 2 using Tukey’s multiple comparisons test from GraphPad version 6. Table S3. Statistical analysis of compound screening studies in Fig. 3 using Tukey’s multiple comparisons test generated from GraphPad version 6. Figure S1. Raw data: Array of phase contrast, fluorescent live/dead images of the breast cancer subgroups MDA-MB-453, MDA-MB-231 and HCC 1937 cultured in 3D perfusion culture. Figure S2. Realtime-Glo™ (RTG) assay optimization for HCC1937 and MDA-MB-231 in the OrganoPlate® for day 0 and day 7 culture. Figure S3. MDA-MB-231 were cultured for 1 day in matrigel prior to 48 h 100 μM cisplatin exposure in the OrganoPlate®. (PPTX 82839 kb) [file 12885_2017_3709_MOESM1_ESM.pptx]

## Slide 1
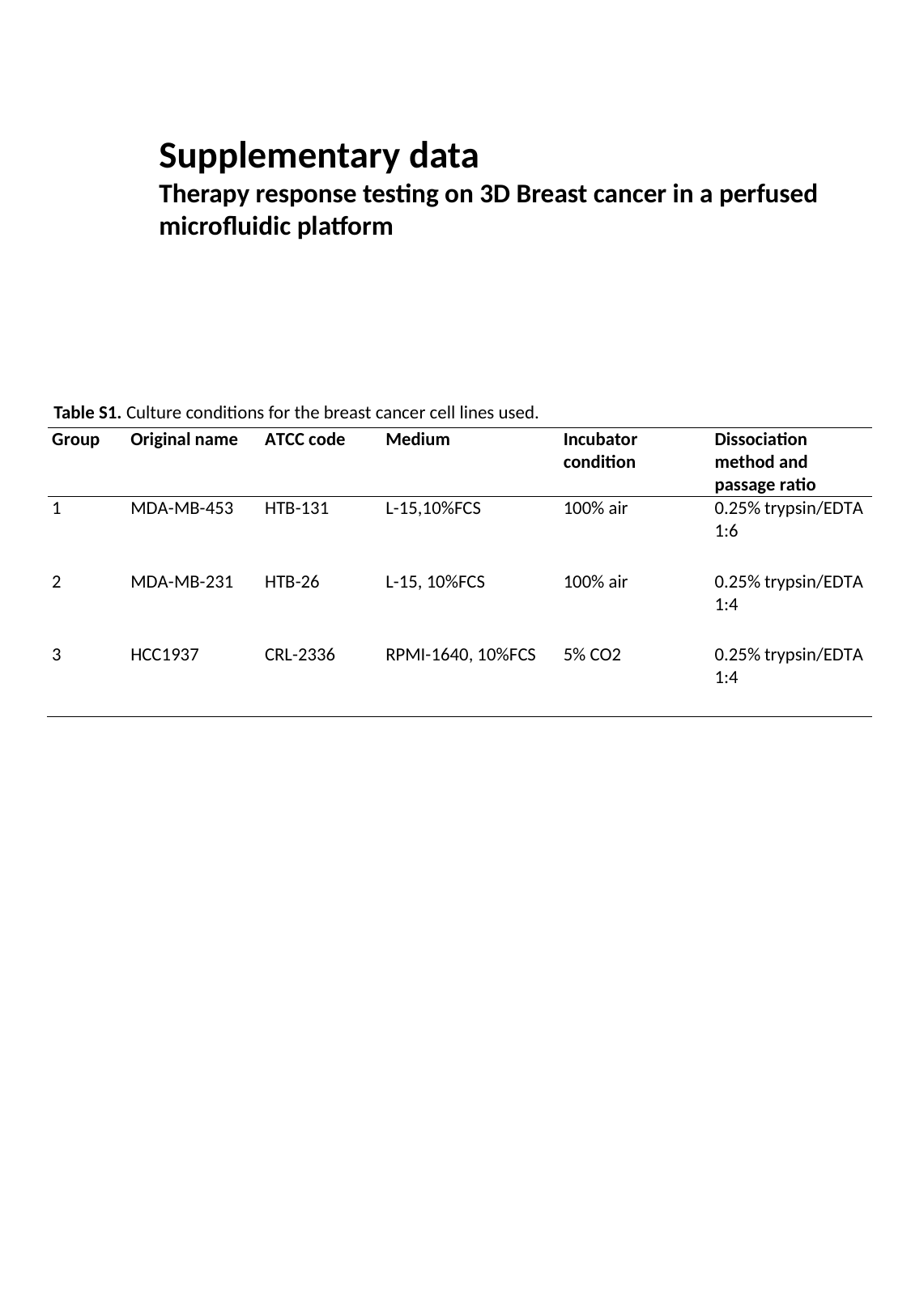

Supplementary data
Therapy response testing on 3D Breast cancer in a perfused microfluidic platform
Table S1. Culture conditions for the breast cancer cell lines used.

## Slide 2
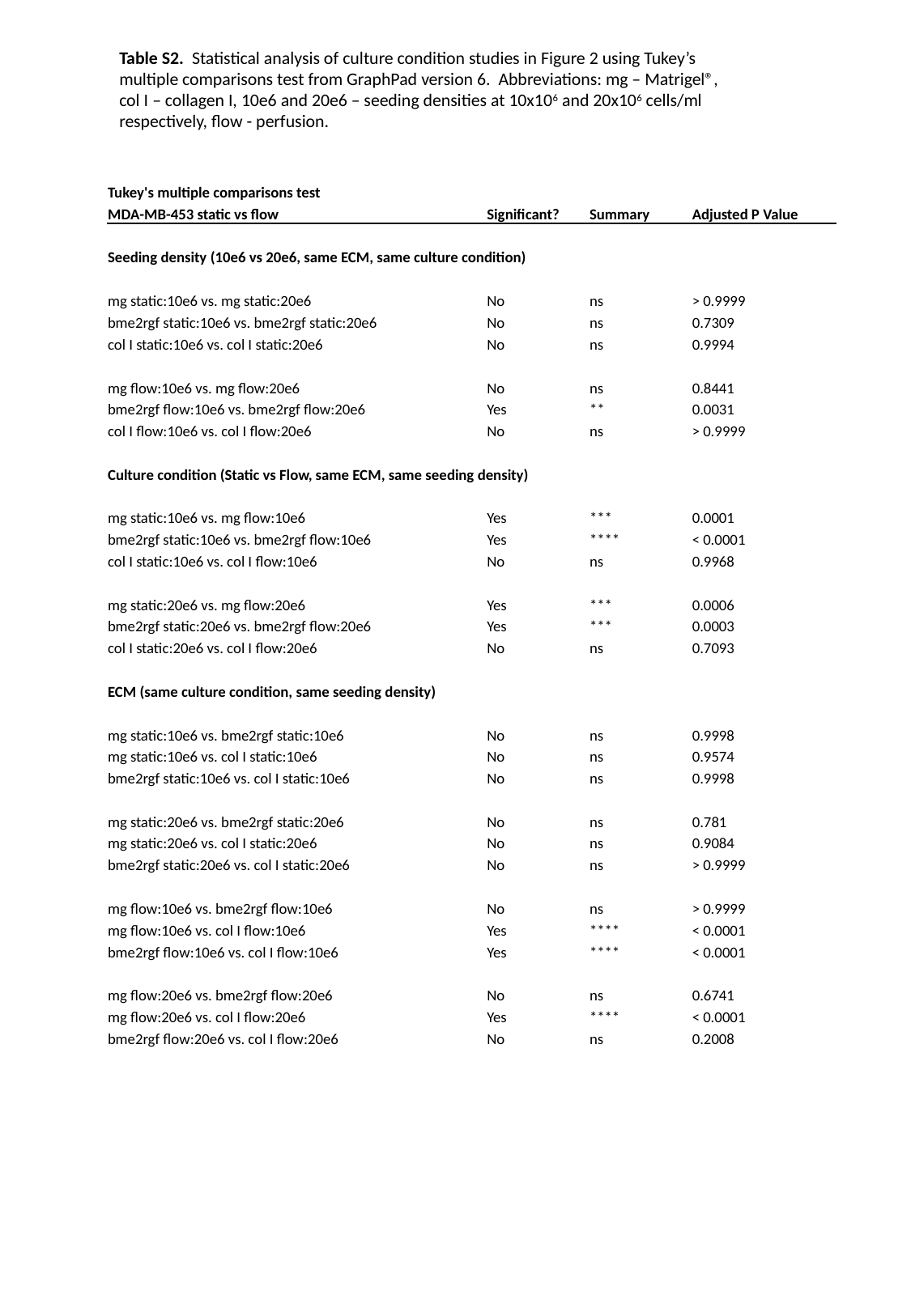

Table S2. Statistical analysis of culture condition studies in Figure 2 using Tukey’s multiple comparisons test from GraphPad version 6. Abbreviations: mg – Matrigel®, col I – collagen I, 10e6 and 20e6 – seeding densities at 10x106 and 20x106 cells/ml respectively, flow - perfusion.
| Tukey's multiple comparisons test | | | |
| --- | --- | --- | --- |
| MDA-MB-453 static vs flow | Significant? | Summary | Adjusted P Value |
| | | | |
| Seeding density (10e6 vs 20e6, same ECM, same culture condition) | | | |
| | | | |
| mg static:10e6 vs. mg static:20e6 | No | ns | > 0.9999 |
| bme2rgf static:10e6 vs. bme2rgf static:20e6 | No | ns | 0.7309 |
| col I static:10e6 vs. col I static:20e6 | No | ns | 0.9994 |
| | | | |
| mg flow:10e6 vs. mg flow:20e6 | No | ns | 0.8441 |
| bme2rgf flow:10e6 vs. bme2rgf flow:20e6 | Yes | \*\* | 0.0031 |
| col I flow:10e6 vs. col I flow:20e6 | No | ns | > 0.9999 |
| | | | |
| Culture condition (Static vs Flow, same ECM, same seeding density) | | | |
| | | | |
| mg static:10e6 vs. mg flow:10e6 | Yes | \*\*\* | 0.0001 |
| bme2rgf static:10e6 vs. bme2rgf flow:10e6 | Yes | \*\*\*\* | < 0.0001 |
| col I static:10e6 vs. col I flow:10e6 | No | ns | 0.9968 |
| | | | |
| mg static:20e6 vs. mg flow:20e6 | Yes | \*\*\* | 0.0006 |
| bme2rgf static:20e6 vs. bme2rgf flow:20e6 | Yes | \*\*\* | 0.0003 |
| col I static:20e6 vs. col I flow:20e6 | No | ns | 0.7093 |
| | | | |
| ECM (same culture condition, same seeding density) | | | |
| | | | |
| mg static:10e6 vs. bme2rgf static:10e6 | No | ns | 0.9998 |
| mg static:10e6 vs. col I static:10e6 | No | ns | 0.9574 |
| bme2rgf static:10e6 vs. col I static:10e6 | No | ns | 0.9998 |
| | | | |
| mg static:20e6 vs. bme2rgf static:20e6 | No | ns | 0.781 |
| mg static:20e6 vs. col I static:20e6 | No | ns | 0.9084 |
| bme2rgf static:20e6 vs. col I static:20e6 | No | ns | > 0.9999 |
| | | | |
| mg flow:10e6 vs. bme2rgf flow:10e6 | No | ns | > 0.9999 |
| mg flow:10e6 vs. col I flow:10e6 | Yes | \*\*\*\* | < 0.0001 |
| bme2rgf flow:10e6 vs. col I flow:10e6 | Yes | \*\*\*\* | < 0.0001 |
| | | | |
| mg flow:20e6 vs. bme2rgf flow:20e6 | No | ns | 0.6741 |
| mg flow:20e6 vs. col I flow:20e6 | Yes | \*\*\*\* | < 0.0001 |
| bme2rgf flow:20e6 vs. col I flow:20e6 | No | ns | 0.2008 |

## Slide 3
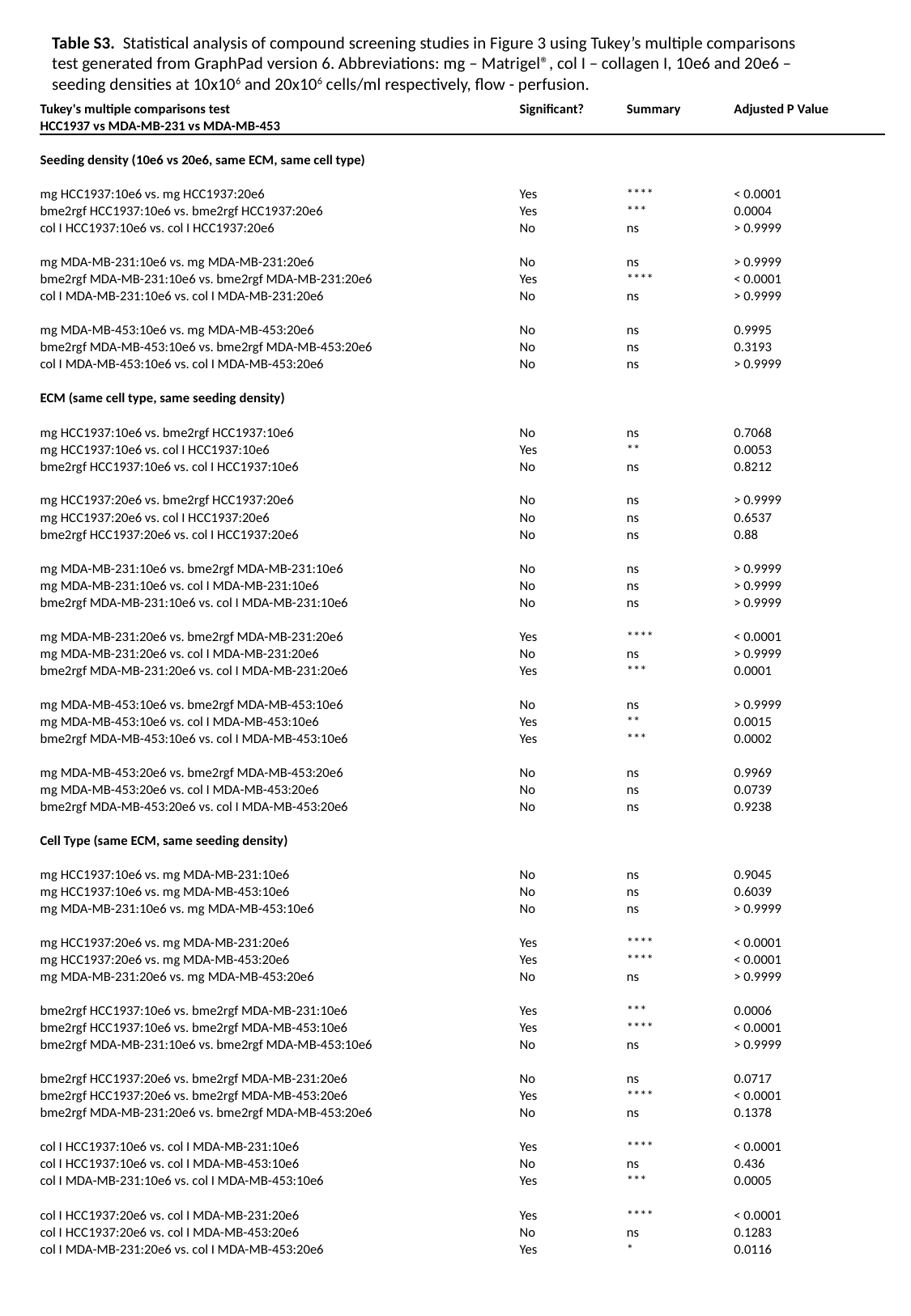

Table S3. Statistical analysis of compound screening studies in Figure 3 using Tukey’s multiple comparisons test generated from GraphPad version 6. Abbreviations: mg – Matrigel®, col I – collagen I, 10e6 and 20e6 – seeding densities at 10x106 and 20x106 cells/ml respectively, flow - perfusion.
| Tukey's multiple comparisons test | Significant? | Summary | Adjusted P Value |
| --- | --- | --- | --- |
| HCC1937 vs MDA-MB-231 vs MDA-MB-453 | | | |
| | | | |
| Seeding density (10e6 vs 20e6, same ECM, same cell type) | | | |
| | | | |
| mg HCC1937:10e6 vs. mg HCC1937:20e6 | Yes | \*\*\*\* | < 0.0001 |
| bme2rgf HCC1937:10e6 vs. bme2rgf HCC1937:20e6 | Yes | \*\*\* | 0.0004 |
| col I HCC1937:10e6 vs. col I HCC1937:20e6 | No | ns | > 0.9999 |
| | | | |
| mg MDA-MB-231:10e6 vs. mg MDA-MB-231:20e6 | No | ns | > 0.9999 |
| bme2rgf MDA-MB-231:10e6 vs. bme2rgf MDA-MB-231:20e6 | Yes | \*\*\*\* | < 0.0001 |
| col I MDA-MB-231:10e6 vs. col I MDA-MB-231:20e6 | No | ns | > 0.9999 |
| | | | |
| mg MDA-MB-453:10e6 vs. mg MDA-MB-453:20e6 | No | ns | 0.9995 |
| bme2rgf MDA-MB-453:10e6 vs. bme2rgf MDA-MB-453:20e6 | No | ns | 0.3193 |
| col I MDA-MB-453:10e6 vs. col I MDA-MB-453:20e6 | No | ns | > 0.9999 |
| | | | |
| ECM (same cell type, same seeding density) | | | |
| | | | |
| mg HCC1937:10e6 vs. bme2rgf HCC1937:10e6 | No | ns | 0.7068 |
| mg HCC1937:10e6 vs. col I HCC1937:10e6 | Yes | \*\* | 0.0053 |
| bme2rgf HCC1937:10e6 vs. col I HCC1937:10e6 | No | ns | 0.8212 |
| | | | |
| mg HCC1937:20e6 vs. bme2rgf HCC1937:20e6 | No | ns | > 0.9999 |
| mg HCC1937:20e6 vs. col I HCC1937:20e6 | No | ns | 0.6537 |
| bme2rgf HCC1937:20e6 vs. col I HCC1937:20e6 | No | ns | 0.88 |
| | | | |
| mg MDA-MB-231:10e6 vs. bme2rgf MDA-MB-231:10e6 | No | ns | > 0.9999 |
| mg MDA-MB-231:10e6 vs. col I MDA-MB-231:10e6 | No | ns | > 0.9999 |
| bme2rgf MDA-MB-231:10e6 vs. col I MDA-MB-231:10e6 | No | ns | > 0.9999 |
| | | | |
| mg MDA-MB-231:20e6 vs. bme2rgf MDA-MB-231:20e6 | Yes | \*\*\*\* | < 0.0001 |
| mg MDA-MB-231:20e6 vs. col I MDA-MB-231:20e6 | No | ns | > 0.9999 |
| bme2rgf MDA-MB-231:20e6 vs. col I MDA-MB-231:20e6 | Yes | \*\*\* | 0.0001 |
| | | | |
| mg MDA-MB-453:10e6 vs. bme2rgf MDA-MB-453:10e6 | No | ns | > 0.9999 |
| mg MDA-MB-453:10e6 vs. col I MDA-MB-453:10e6 | Yes | \*\* | 0.0015 |
| bme2rgf MDA-MB-453:10e6 vs. col I MDA-MB-453:10e6 | Yes | \*\*\* | 0.0002 |
| | | | |
| mg MDA-MB-453:20e6 vs. bme2rgf MDA-MB-453:20e6 | No | ns | 0.9969 |
| mg MDA-MB-453:20e6 vs. col I MDA-MB-453:20e6 | No | ns | 0.0739 |
| bme2rgf MDA-MB-453:20e6 vs. col I MDA-MB-453:20e6 | No | ns | 0.9238 |
| | | | |
| Cell Type (same ECM, same seeding density) | | | |
| | | | |
| mg HCC1937:10e6 vs. mg MDA-MB-231:10e6 | No | ns | 0.9045 |
| mg HCC1937:10e6 vs. mg MDA-MB-453:10e6 | No | ns | 0.6039 |
| mg MDA-MB-231:10e6 vs. mg MDA-MB-453:10e6 | No | ns | > 0.9999 |
| | | | |
| mg HCC1937:20e6 vs. mg MDA-MB-231:20e6 | Yes | \*\*\*\* | < 0.0001 |
| mg HCC1937:20e6 vs. mg MDA-MB-453:20e6 | Yes | \*\*\*\* | < 0.0001 |
| mg MDA-MB-231:20e6 vs. mg MDA-MB-453:20e6 | No | ns | > 0.9999 |
| | | | |
| bme2rgf HCC1937:10e6 vs. bme2rgf MDA-MB-231:10e6 | Yes | \*\*\* | 0.0006 |
| bme2rgf HCC1937:10e6 vs. bme2rgf MDA-MB-453:10e6 | Yes | \*\*\*\* | < 0.0001 |
| bme2rgf MDA-MB-231:10e6 vs. bme2rgf MDA-MB-453:10e6 | No | ns | > 0.9999 |
| | | | |
| bme2rgf HCC1937:20e6 vs. bme2rgf MDA-MB-231:20e6 | No | ns | 0.0717 |
| bme2rgf HCC1937:20e6 vs. bme2rgf MDA-MB-453:20e6 | Yes | \*\*\*\* | < 0.0001 |
| bme2rgf MDA-MB-231:20e6 vs. bme2rgf MDA-MB-453:20e6 | No | ns | 0.1378 |
| | | | |
| col I HCC1937:10e6 vs. col I MDA-MB-231:10e6 | Yes | \*\*\*\* | < 0.0001 |
| col I HCC1937:10e6 vs. col I MDA-MB-453:10e6 | No | ns | 0.436 |
| col I MDA-MB-231:10e6 vs. col I MDA-MB-453:10e6 | Yes | \*\*\* | 0.0005 |
| | | | |
| col I HCC1937:20e6 vs. col I MDA-MB-231:20e6 | Yes | \*\*\*\* | < 0.0001 |
| col I HCC1937:20e6 vs. col I MDA-MB-453:20e6 | No | ns | 0.1283 |
| col I MDA-MB-231:20e6 vs. col I MDA-MB-453:20e6 | Yes | \* | 0.0116 |

## Slide 4
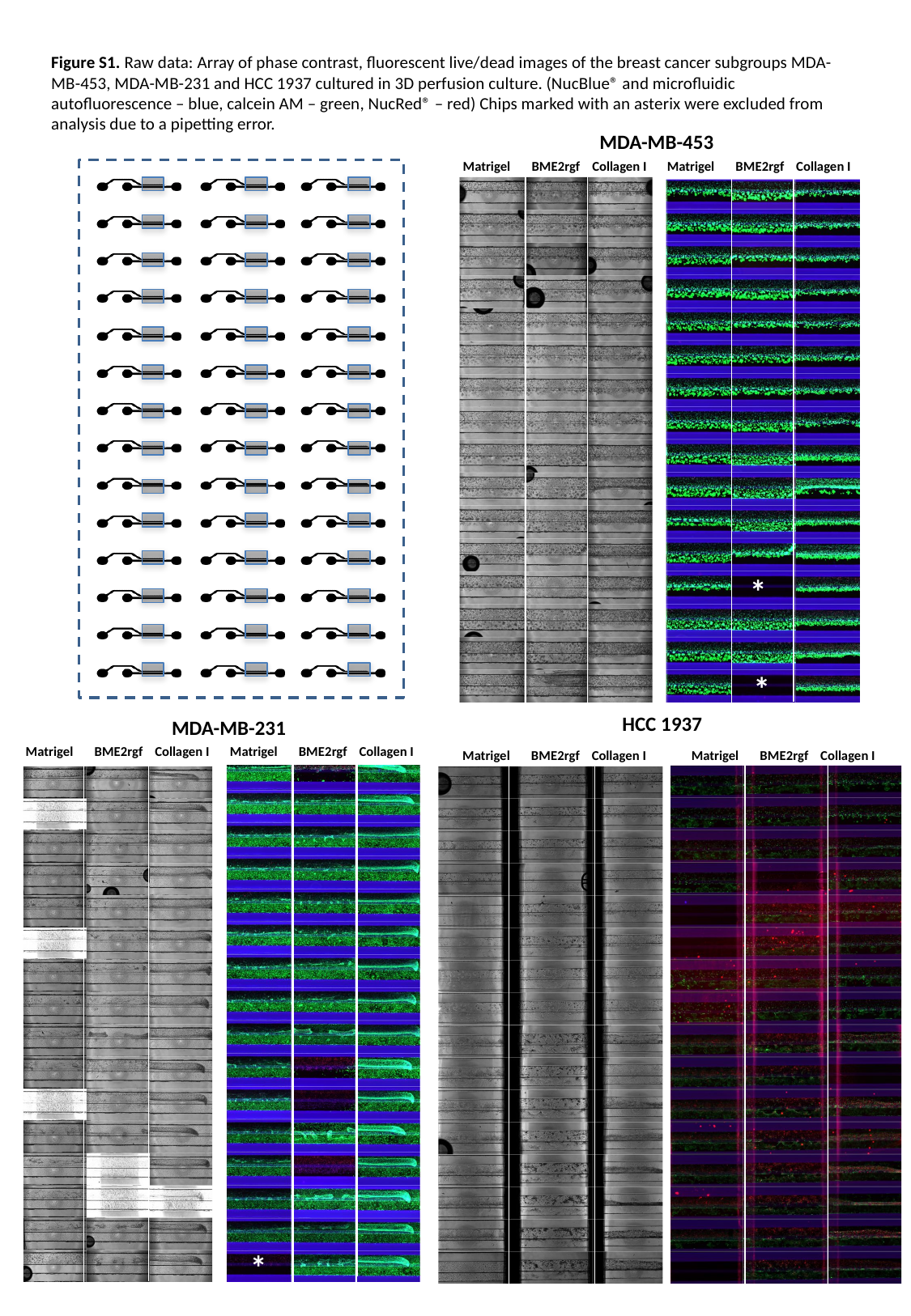

Figure S1. Raw data: Array of phase contrast, fluorescent live/dead images of the breast cancer subgroups MDA-MB-453, MDA-MB-231 and HCC 1937 cultured in 3D perfusion culture. (NucBlue® and microfluidic autofluorescence – blue, calcein AM – green, NucRed® – red) Chips marked with an asterix were excluded from analysis due to a pipetting error.
MDA-MB-453
 Matrigel BME2rgf Collagen I
 Matrigel BME2rgf Collagen I
*
*
HCC 1937
MDA-MB-231
 Matrigel BME2rgf Collagen I
 Matrigel BME2rgf Collagen I
 Matrigel BME2rgf Collagen I
 Matrigel BME2rgf Collagen I
*

## Slide 5
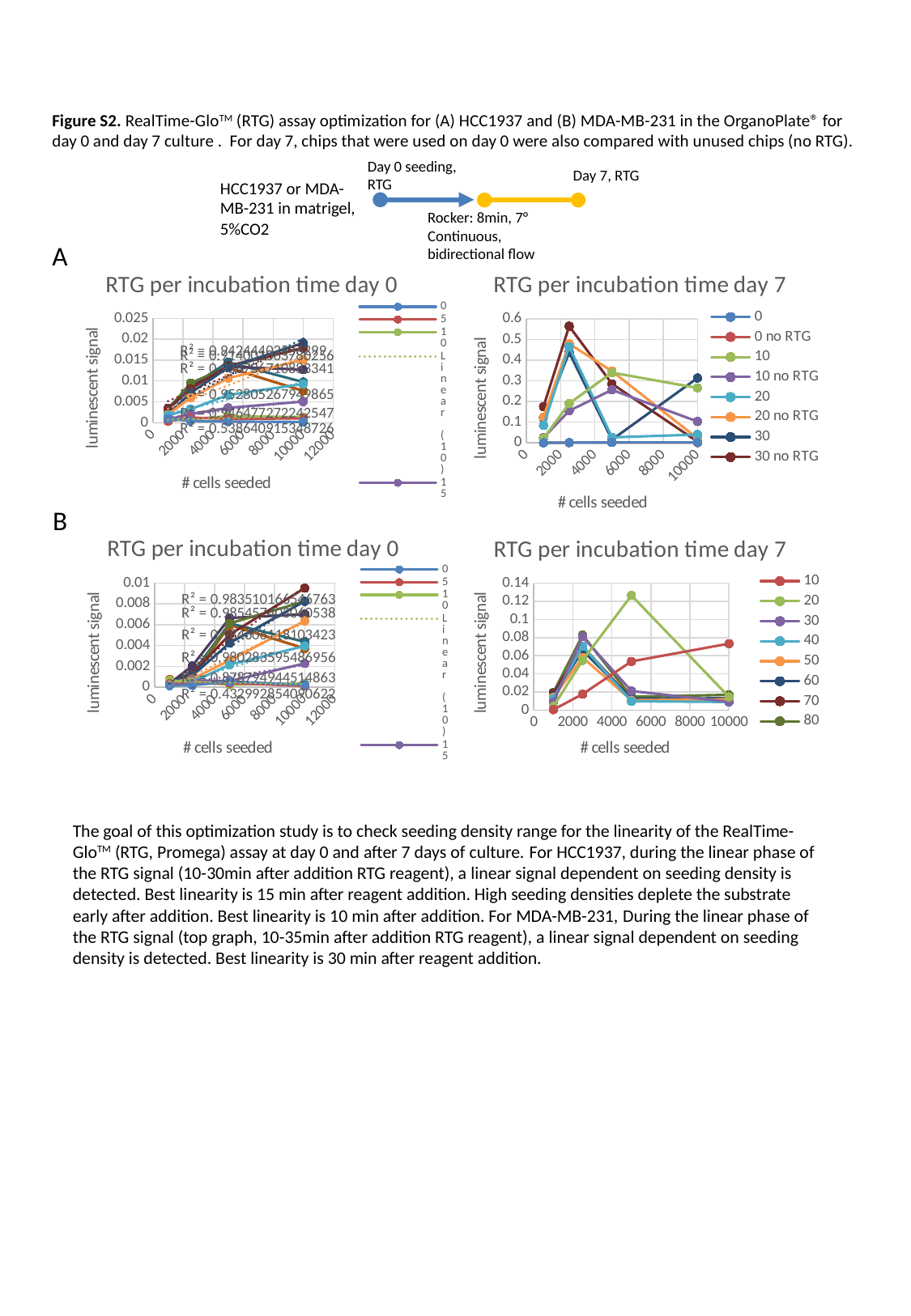

Figure S2. RealTime-GloTM (RTG) assay optimization for (A) HCC1937 and (B) MDA-MB-231 in the OrganoPlate® for day 0 and day 7 culture . For day 7, chips that were used on day 0 were also compared with unused chips (no RTG).
Day 0 seeding, RTG
Day 7, RTG
HCC1937 or MDA-MB-231 in matrigel, 5%CO2
Rocker: 8min, 7°
Continuous, bidirectional flow
A
### Chart: RTG per incubation time day 0
| Category | | | | | | | | | | | | | |
|---|---|---|---|---|---|---|---|---|---|---|---|---|---|
### Chart: RTG per incubation time day 7
| Category | | | | | | | | |
|---|---|---|---|---|---|---|---|---|B
### Chart: RTG per incubation time day 0
| Category | | | | | | | | | | | | | |
|---|---|---|---|---|---|---|---|---|---|---|---|---|---|
### Chart: RTG per incubation time day 7
| Category | | | | | | | | |
|---|---|---|---|---|---|---|---|---|The goal of this optimization study is to check seeding density range for the linearity of the RealTime-GloTM (RTG, Promega) assay at day 0 and after 7 days of culture. For HCC1937, during the linear phase of the RTG signal (10-30min after addition RTG reagent), a linear signal dependent on seeding density is detected. Best linearity is 15 min after reagent addition. High seeding densities deplete the substrate early after addition. Best linearity is 10 min after addition. For MDA-MB-231, During the linear phase of the RTG signal (top graph, 10-35min after addition RTG reagent), a linear signal dependent on seeding density is detected. Best linearity is 30 min after reagent addition.

## Slide 6
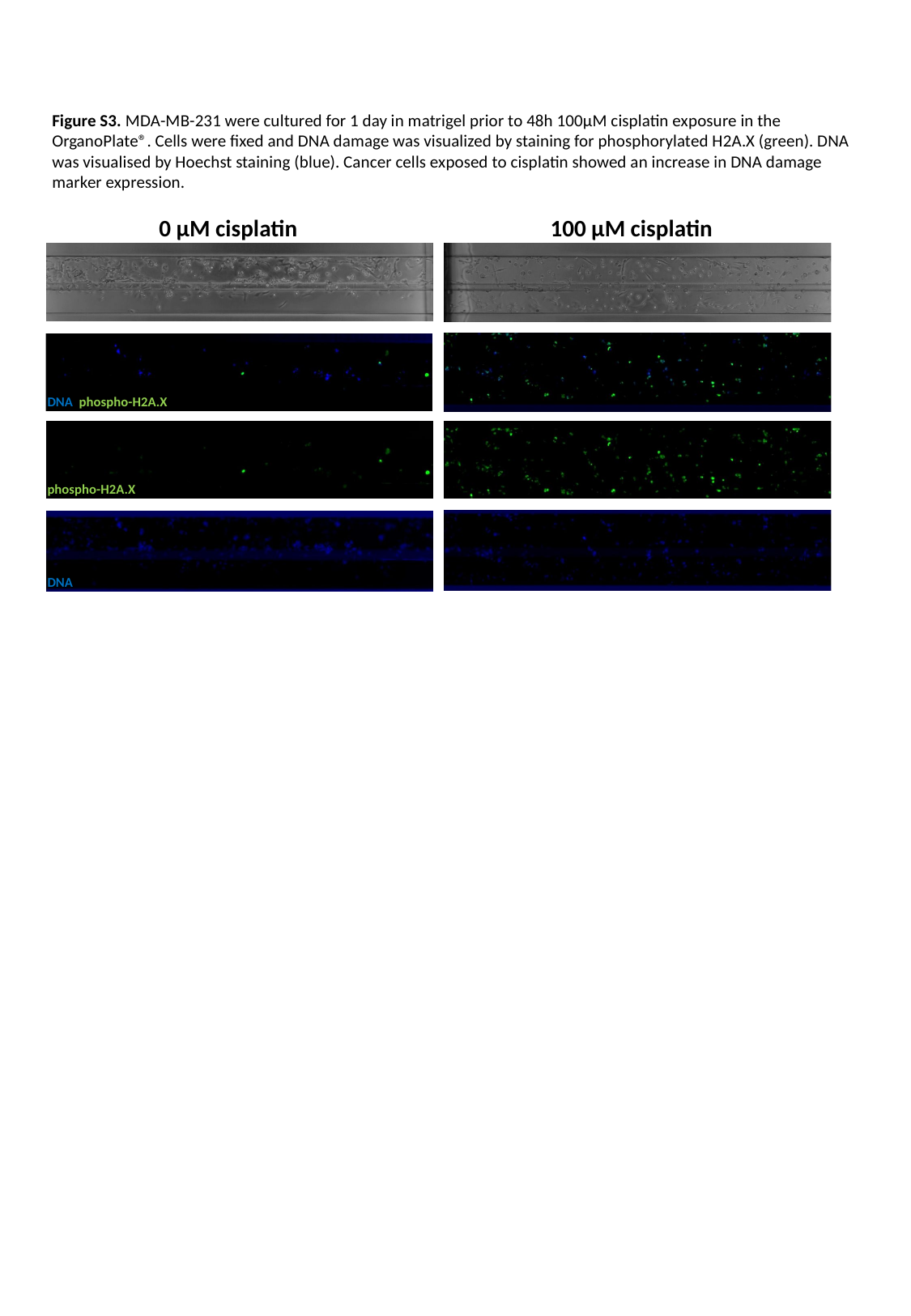

Figure S3. MDA-MB-231 were cultured for 1 day in matrigel prior to 48h 100µM cisplatin exposure in the OrganoPlate®. Cells were fixed and DNA damage was visualized by staining for phosphorylated H2A.X (green). DNA was visualised by Hoechst staining (blue). Cancer cells exposed to cisplatin showed an increase in DNA damage marker expression.
0 µM cisplatin
100 µM cisplatin
DNA phospho-H2A.X
phospho-H2A.X
DNA
